# Hypoxia Promotes Gastric Cancer Malignancy Partly through the HIF-1α Dependent Transcriptional Activation of the Long Non-coding RNA GAPLINC

**DOI:** 10.3389/fphys.2016.00420

**Published:** 2016-09-27

**Authors:** Lei Liu, Xihe Zhao, Huawei Zou, Rubing Bai, Keyu Yang, Zhong Tian

**Affiliations:** ^1^General Surgery Department, Shengjing Hospital, China Medical University Shenyang, China; ^2^Oncology Department, Shengjing Hospital, China Medical University Shenyang, China; ^3^General Surgery Department, The Forth Hospital, China Medical University Shenyang, China

**Keywords:** hypoxia, HIF-1α, lncRNA, GAPLINC, gastric cancer

## Abstract

Hypoxia-inducible factor (HIF) activates the transcription of genes involved in cancer progression. Recently, HIF was reported to regulate the transcription of non-coding RNAs. Here, we show that the transcription of a long non-coding RNA (lncRNA), Gastric Adenocarcinoma Associated, Positive CD44 Regulator, Long Intergenic Non-Coding RNA (GAPLINC), is directly activated by HIF-1α in gastric cancer (GC). GAPLINC was overexpressed in GC tissues and promoted tumor migration and invasive behavior. GAPLINC overexpression was associated with poor prognosis in GC patients. Luciferase reporter assays and chromatin immunoprecipitation assays confirmed that HIF-1α binds to the promoter region of GAPLINC and activates its transcription. GAPLINC knockdown inhibited hypoxia-induced tumor proliferation *in vivo*. Taken together, our results identified a novel role for HIF transcriptional pathways in GC tumorigenesis mediated by the regulation of the lncRNA GAPLINC, and suggest GAPLINC as a novel therapeutic target for reversing chemoradioresistance and prolonging survival.

## Introduction

Solid tumor cells are frequently exposed to low levels of oxygen, and activation of hypoxia-related pathways is a common feature of many types of cancers (Harris, 2002; Pouysségur et al., 2006). Cells stimulate angiogenesis and metabolic alterations to adapt to hypoxia for survival and progression, and these changes lead to an aggressive tumor phenotype, resistance to chemotherapy, and poor clinical outcomes (Nordgren and Tavassoli, 2011).

The human gastric mucosa is exposed to a hypoxic environment (Payne and Bowen, 1981; Tarnawski et al., 2007), and the extent of hypoxia is obvious in gastric cancer (GC). The hypoxic microenvironment in the tumor center and aberrant expression of oncogenes facilitate pro-oncogenic effects (Tarnawski et al., 2007; Matsumoto et al., 2009; Wang et al., 2011). One of the major factors involved in hypoxic signaling pathways is hypoxia-inducible factor (HIF). HIF-1 is a heterodimeric transcription factor composed of an α subunit and a β subunit. HIF-1α is the active subunit of HIF-1 and the predominant mediator of adaptation to hypoxia. It can recognize and bind to hypoxia response elements (HREs: 5′-A/GCGTG-3′) (Shinohara and Maity, 2009), activating the transcription of many genes to mediate pro-oncogenic events including angiogenesis (Nordgren and Tavassoli, 2011; Li J. et al., 2014), inflammatory responses (Lee et al., 2014), and growth factor stimulation (Schito et al., 2012). HIF-1α controls central metastasis-associated pathways (Branco-Price et al., 2012; Ji, 2014). In GC, HIF-1α exerts a tumorigenic effect by promoting cell growth, vessel maturation, and cell invasion (Stoeltzing et al., 2004).

Integrative genomic studies showed that over 10,000 long non-coding RNAs (lncRNAs) are encoded by the human genome (Lee, 2012; Batista and Chang, 2013). Despite rapid progress in screening cancer-related lncRNAs, few lncRNAs associated with GC have been characterized, including H19 (Li H. et al., 2014), HOTAIR (Zhang et al., 2015), and ANRIL (Zhang et al., 2014). GAPLINC, a newly identified 924-bp lncRNA (Hu et al., 2014) was predicted to contain HREs in the promoter region by DataBase of Transcriptional Start Sites (DBTSS). It is deregulated in GC and associated with copy number variations or oncogenic transcription factors. GAPLINC regulates CD44 as a molecular sponge for miR-211-3p and enhances tumor migration and invasion. GAPLINC is therefore considered valuable for the diagnosis and prognosis prediction of GC.

Here, we investigated the expression of GAPLINC under conditions of hypoxia and normoxia. We found that GAPLINC is regulated by hypoxia in the GC cell lines MKN45 and SGC7901. Hypoxic induction of GAPLINC by HIF-1α leads to enhanced cell proliferation, migration, and invasion, and decreased apoptosis. Our results showed that hypoxia-induced GAPLINC accelerates tumor progression in GC.

## Materials and methods

### Clinical specimens

Tissues were obtained from surgical resection in Shengjing Hospital of China Medical University, including 18 cases of normal gastric tissues (NGT), 17 cases of highly differentiated gastric adenocarcinoma (HDAC) tissues, and 16 cases of poorly differentiated gastric adenocarcinoma (PDAC) tissues. NGTs were obtained from normal gastric perforation or benign ulcer surgeries away from the lesion. Tumor specimens were collected from general gastric cancer radical surgeries. Fresh tissues were immediately stored in liquid nitrogen after surgical removal. All patients provided written informed consent. The experiment was approved by the ethics committee of China Medical University Shengjing Hospital.

### Cell culture

Human gastric mucosa cell lines GEF-1, HFE-145, human gastric cancer cell lines MKN45, SGC7901, and human embryonic kidney cell line HEK-293T were obtained from the Shanghai Institutes for Biological Sciences Cell Resource Center. HFE-145, MKN45 cells, and HEK-293T cells were maintained in DMEM (Thermo Fisher Scientific Inc, MA, USA) containing 10% fetal bovine serum (FBS, Life Technologies Corporation, Paisley, UK). SGC7901 and GES-1 cells were cultured in RPMI-1640 (Sigma-Aldrich, St Louis, MO, USA) containing 10% FBS. Cells were incubated in a humidified incubator at 37°C with 5% CO_2_ in an atmosphere of either normoxia (21% oxygen), or hypoxia (1% O_2_, 5% CO_2_, and 94% N_2_ gas mixture) for 24 h.

### Plasmid construction and DNA transfection

HIF-1α or HIF-2α subunits were suppressed using siRNAs (Elvidge et al., 2006). The shRNA against human GAPLINC (18p11.31) were constructed. MKN45 and SGC7901 cell lines were transfected with GAPLINC shRNA, and named as shGAPLINC cells. For the negative control, empty vectors were used and named as NC. Opti-MEM I and Lipofectamine 3000 reagents (Invitrogen, CA, USA) were used according to the manufacturer's instructions. Stable cell lines were created through the selection by means of Geneticin (G418; Sigma-Aldrich, St Louis, MO, USA), G418-resistant clones were obtained after 3–4 weeks. The transfection efficacy was assessed by quantitative real-time PCR (qRT-PCR).

### RNA extraction and real-time quantitative PCR

After extraction of total RNA using Trizol (Life Technologies Corporation), RNA concentration and quality were evaluated by NanoDrop spectrophotometer (ND-100, Thermo, USA). SYBR Premix Ex Taq assays of GAPLINC and GAPDH (Applied Biosystems, Foster City, CA, USA) were used for GAPLINC qRT-PCR detection. For each assay, a standard curve of threshold cycle (C_T_) value vs. log input standard cDNA was constructed. Fold changes were normalized and calculated using the relative quantification (2^−ΔΔCt^) method.

### Western blot analysis

HIF-1α, HIF-2α content was detected by western blot. Cell homogenate protein was prepared using loading buffer, RIPA lysis buffer as well as protease inhibitor (Beyotime Institute of Biotechnology, Jiangsu, China). Cell homogenate was then boiled for 5 min. Samples were centrifuged at 17,000 rpm, 4°C. The concentrations of proteins were measured using the BCA protein assay kit (Beyotime Institute of Biotechnology).

Protein samples of interest went electrophoresis and then were transferred to polyvinylidene fluoride membrane. Membrane was blocked in TBST (Tween Tris-buffered saline) buffer containing 5% low fat milk powder, then incubated with primary antibodies respectively at 4°C overnight. Primary antibodies included HIF-1α (diluted at 1:1000, Abcam, Cambridge, UK), HIF-2α (1:500, Abcam), Bcl-2 (1:2000, Proteintech Group, Chicago, USA), Bax (1:3000, Proteintech Group), CD44 (1:1000, Proteintech Group), TGFBR2 (1:500, Abcam), GAPDH (1:5000, Cell Signaling, Beverly, MA, USA). Then, membranes were incubated with corresponding HRP-polymerization secondary antibodies diluted at 1:5000 for 2 h at room temperature. Protein bands were visualized by ECL detection system, and ChemImager5500V2.03 scanning software. The integrated light density values (IDV) were calculated with software FluorChem2.0.

### Cell viability assay

CCK8 reagent (Beyotime institute of biotechnology) and 5-ethynyl-2′-deoxyuridine (EdU) incorporation assay kit (Life Technologies Corporation) were used for cell proliferation ability detection. For CCK8 assay, MKN45 and SGC7901 cells were seeded in 96 well-plates (Corning, NY, USA) at the logarithmic growth phase, at a density of 2000/well each group. Two days later, 10 μl of CCK8 reagent was added into the culture medium and then incubated for 2 h. The absorbance at 450 nm was recorded.

For EdU incorporation assay, cells were cultured in medium with 10 μM of EdU each well and incubated for 2 h at 37°C. After fixing with 4% formaldehyde, cells were incubated with a Click-iT EdU Kit (Life Technologies Corporation), then stained with Hoechst 33342 and visualized using a fluorescence microscope (Olympus, Tokyo, Japan). The EdU incorporation rate was measured using the ratio of EdU positive cells (green cells) to total Hoechst 33342 positive cells (blue cells). Scale bar represented for 20 μm.

### Cell apoptosis assay

Cell apoptosis rate was measured using the Annexin V-PI/FITC kit (BD Biosciences, NJ, USA). Cells were resuspended into single cell suspension after washed with ice cold PBS. Then cells were collected with centrifugation and stained with Annexin V-PI and FITC for 15 min away from light. After that cells went for flow cytometry (FACScan, BD) detection, total cells and apoptotic cells which were stained with fluorescent dye were counted. Apoptosis ratio was analyzed using BD Accuri C6 software.

### Cell migration and invasion assay

In cell migration and invasion assays, the 24-well plates and transwell chambers with 8 μm size pore polycarbonate membranes (Corning, NY, USA) were used. Cells were re-suspended in serum-free medium, and then seeded on the upper chamber. Matrigel solution (BD, Franklin Lakes, NJ, USA) was precoated before cell seeding for cell invasion assay. The serum-containing medium in the 24-well plates induced cells to migrate and invade. After incubated at 37°C for 48 h, the cells on the upper chamber were stripped off with a swab. Then cells on the lower membrane were fixed with the mixture of glacial acetic acid and methanol (3:1), and then stained with 20% Giemsa solution overnight. Five vision fields were randomly chosen and cells number was recorded for each well.

### Luciferase reporter assay

By screening the promoter region of GAPLINC, two binding motifs (CACGC; Ren et al., 2014 and ACGTG) of HIF-1α were found. To determine the responsive HIF-1α-binding sites in the human GAPLINC promoter, promoter activities were measured using Dual-Luciferase Reporter Assay System. Human full-length HIF-1α sequence was cloned into pEX3 vector (GenePharma). pEX3-HIF-1α and its empty vector were transfected into MKN45 and SGC7901 cells. Renilla promoters were co-transfected as an internal control. Firefly luciferase activity was normalized to renilla luciferase activity for each individual analysis.

### Chromatin immunoprecipitation

Chromatin immunoprecipitation (ChIP) assay was carried out with Simple ChIP. Enzymatic Chromatin IP Kit (Cell Signaling Technology, Danvers, Massachusetts, USA). Cells were cross-linked with 1% paraformaldehyde, and then harvested in lysis buffer containing phenylmethane sulfonyl fluoride. Chromatin was cleaved into fragments (200–1000 bps). The 1000 bps upstream region of the putative HIF-1α binding sites was used as a negative control in ChIP assay. Lysates (2%) were used as input control. The rest lysates were immunoprecipitated with normal mouse IgG or HIF-1α antibody.

The DNA was extracted and amplified by PCR. The forward and the reverse primers were: PCR1 Forward: 5′-CAACCCTAATGGG ACGTGTTA-3′, Reverse: 5′-CAAACAGAATCA CCCCATGA-3′; PCR2 Forward: 5′-TAATCCCAG CTACTTGGGAGG-3′, Reverse: 5′-ATTCTTGCC CAGGCTGGAAT-3′, which covered a potential HRE (hypoxia response elements). The primers of the control region were: Forward: 5′-GGAAAATAA AACCATGGAGGG-3′ Reverse: 5′-CGAGAT TGAGCCACTGCACT-3′.

### Subcutaneous xenograft model in nude mice

For the *in vivo* study, xenograft experiments were performed in balb/c nude mice. Mice were purchased from BEIJING HFK BIOSCIENCE Co. LTD and raised under specific pathogen-free condition, under the guidance of the laboratory animal center guideline. All process involving animals was subjected to approval by the Research Animal Care and Use Committee of Shengjing Hospital. Cells were subcutaneously implanted into the right flanks of mice, at density of 1 × 10^6^ cells/mouse. Every 5 days, tumor volumes were measured. Once tumors reached 150 mm^3^, either bevacizumab (10 mg/kg every 3 days) or vehicle control PBS (Phosphate Buffered Saline) were intraperitoneal injected to the mice. Tumor volume (mm^3^) = length × width^2^/2.

### Statistical analysis

All data were presented as means ± standard deviation (SD) and derived from at least three independent experiments. All statistical analyses were performed using SPSS 18.0 statistical software with the Student's *t*-test. Probability *P* < 0.05 was considered as statistically significant. The spearman rank correlation test was used for the association between different variables analyzation. For survival analysis, Mantel-cox test was used.

## Results

### HIF-1α and GAPLINC are upregulated in GC tissues and cell lines and associated with poor patient prognosis

**Figure 1:**
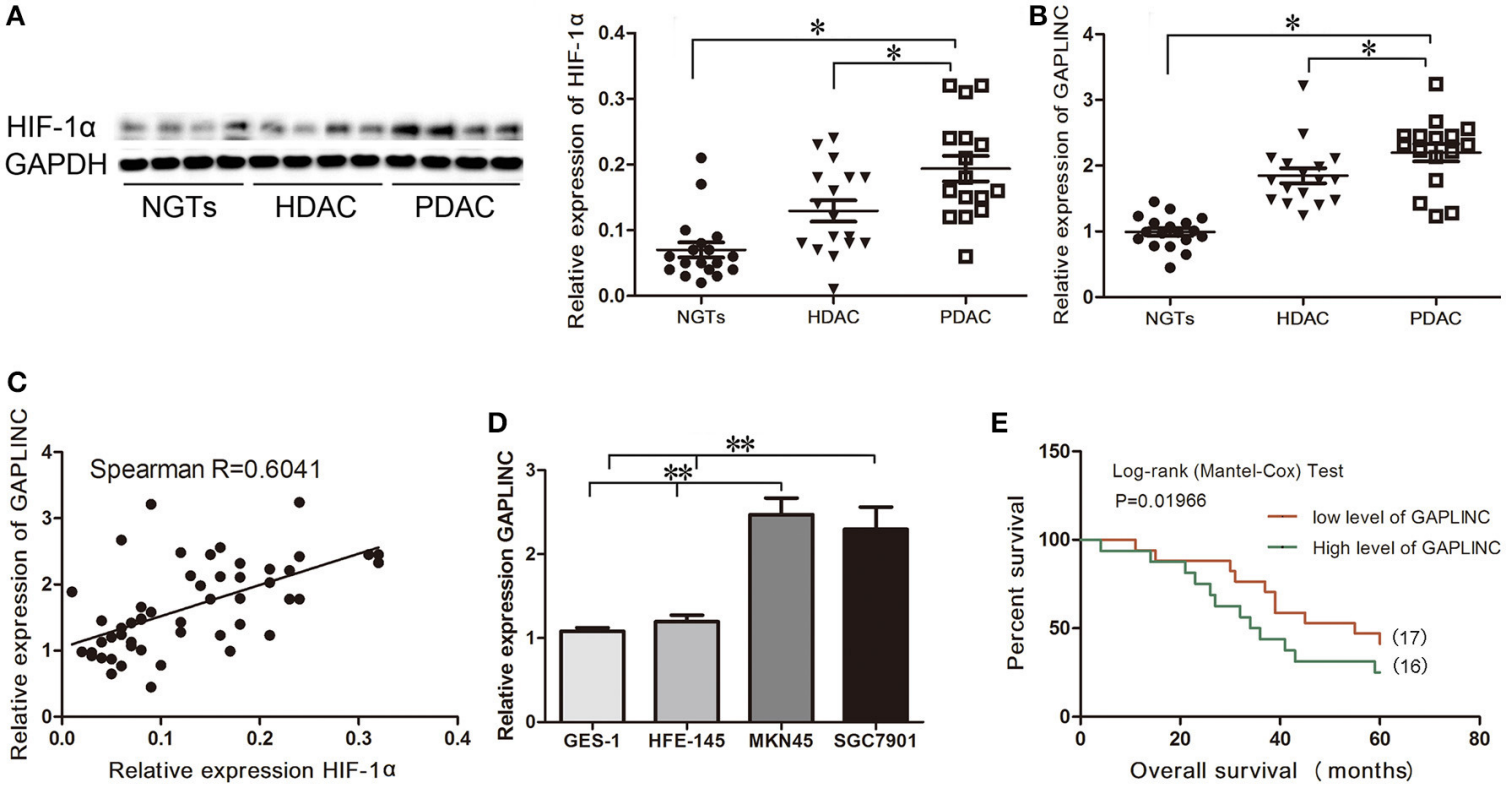
**Correlation between HIF-1α protein expression and GAPLINC expression**. GAPLINC overexpression was associated with adverse clinical outcomes in patients with gastric cancer (GC). Data represent the mean ± SD. **(A)** HIF-1α was highly expressed in human gastric tissues (*n* = 3 each group). **(B)** The endogenous expression of GAPLINC was determined by qRT-PCR in control gastric tissues, highly differentiated gastric adenocarcinoma tissues, and poorly differentiated gastric adenocarcinoma tissues. **(C)** Spearman correlation between HIF-1α protein expression and GAPLINC expression (*R* = 0.6041). **(D)** GAPLINC was highly expressed in GCcell lines compared to normal gastric mucosa cell lines. **(E)** Overall survival (OS) of patients with GAPLINC-low expression (17 cases) and high expression (16 cases). GAPLINC expressions were normalized to the ratios of expressions in tissues to one specimen picked randomly in NGTs (ranged from 1.23 to 3.24, and the cut off point was 2.03). The Mantel-Cox test was used to compare OS after surgery. The GAPLINC-low expression group had longer OS (^*^P < 0.05, ^**^P < 0.01).

A recent study reported that the lncRNA GAPLINC is upregulated in human GC (Hu et al., 2014). Assessment of HIF-1α expression showed that it was markedly upregulated under conditions of hypoxia in GC specimens (Figure 1). Immunoblot analysis showed that HIF-1α was highly expressed in gastric adenocarcinoma, especially in poorly-differentiated tumors, compared with NGT (*P* < 0.05, Figure 1A). qRT-PCR results showed that the pattern of expression of GAPLINC was similar to that of HIF-1α (*P* < 0.05, Figure 1B). Spearman correlation analysis showed a positive correlation between GAPLINC and HIF-1α expression (*R* = 0.6041, *P* < 0.05, Figure 1C). We therefore examined the association between HIF-1α and GAPLINC in further detail. GAPLINC was expressed at higher levels in the GC cell lines MKN45 and SGC7901 than in the gastric mucosa cell lines GES-1 and HFE-145 (Figure 1D). Overall survival analysis indicated that GAPLINC overexpression was associated with poor prognosis in GC patients (*P* < 0.05, Figure 1E).

### GAPLINC was upregulated under conditions of hypoxia

**Figure 2:**
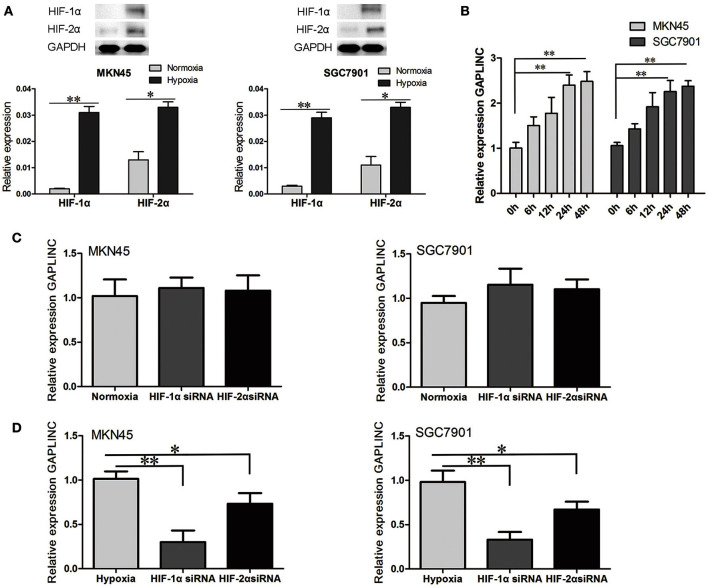
**GAPLINC expression was induced under hypoxia mainly by HIF-1α**. Data represent the mean ± SD. **(A)** HIF-1α and HIF-2α were upregulated in MKN45 and SGC7901 cells under hypoxic conditions. **(B)** GAPLINC was upregulated under hypoxia in MKN45 and SGC7901 cells. **(C)** qPCR analysis of the normoxic expression of GAPLINC in MKN45 and SGC7901 cells following transfection with control siRNA, HIF-1α siRNA, or HIF-2α siRNA. **(D)** The same experiments were performed in hypoxia, demonstrating marked dependence on HIF-1α (^*^P < 0.05, ^**^P < 0.05).

Accumulating evidence indicates that the hypoxic microenvironment provides an important niche for drug-resistant cells (Brown, 1990; Trédan et al., 2007; Lin et al., 2011). HIF, as a finely regulated transcriptional factor, could stably exist in the hypoxic environment in MKN45 and SGC7901 cells (Figure 2A). To investigate the effect of hypoxia on GAPLINC expression, GAPLINC levels were monitored under conditions of normoxia and hypoxia. GAPLINC expression was gradually upregulated in both cell lines in a hypoxic atmosphere, and after 24 h the difference became significant (Figure 2B, *P* < 0.01). Knockdown of the two active subunits of HIF (HIF-1α and HIF-2α) did not affect GAPLINC expression under normoxia, whereas GAPLINC was markedly downregulated under hypoxia. The reduction was more obvious in the siHIF-1α group (Figures 2C,D).

### Hypoxic induction of GAPLINC accelerated tumor cell proliferation, migration, and invasion and inhibited cell apoptosis

**Figure 3:**
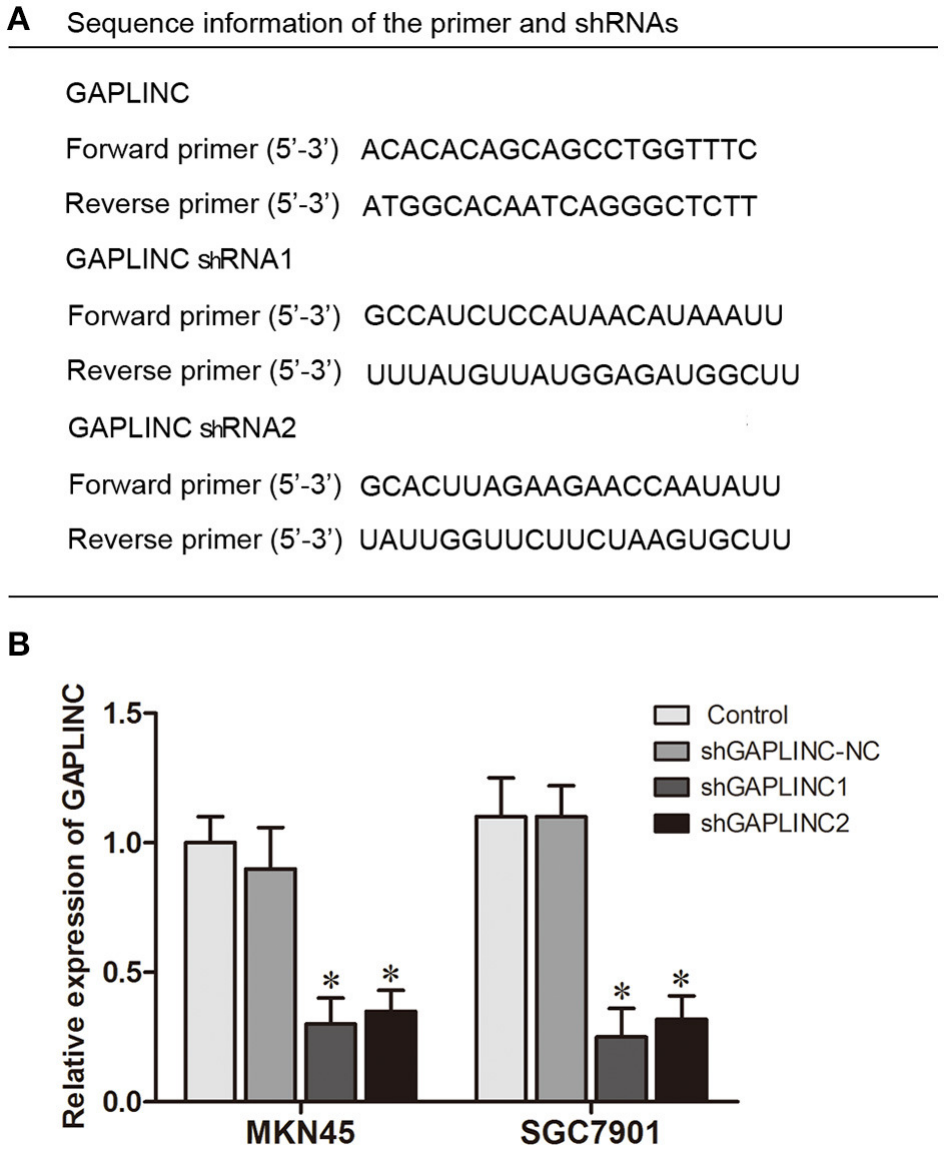
**Knockdown of GAPLINC with shRNAs. (A)** Sequence information of the primers and shRNAs used. **(B)** Silencing efficiency of transfection using two shRNAs. The results were derived from at least three different experiments (^*^P < 0.05).

**Figure 4:**
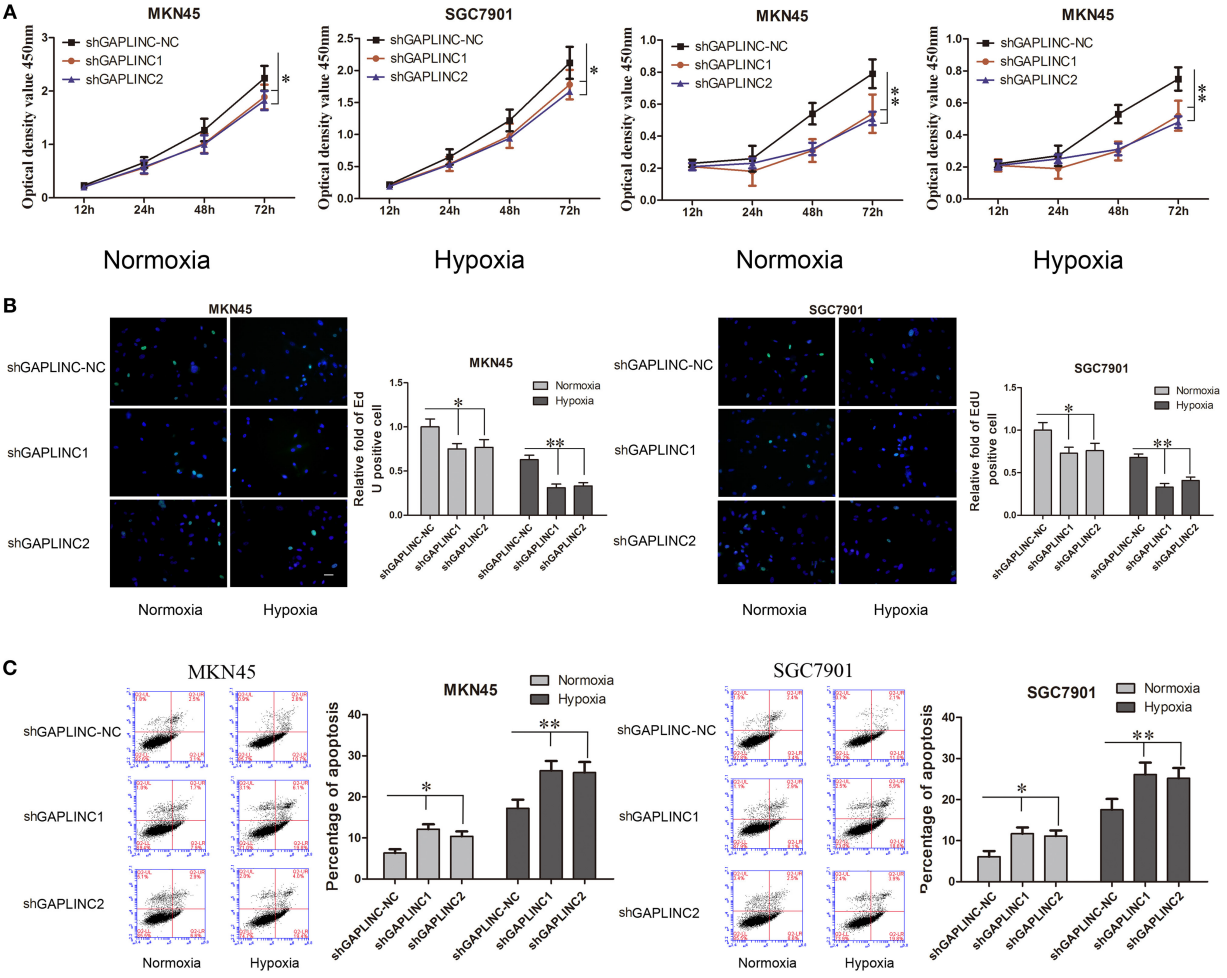
**GAPLINC sustained tumor cell growth and decreased cell apoptosis in MKN45 and SGC7901 cells especially under hypoxic conditions**. Values represent the mean ± SD. **(A)** Cell proliferation rates detected using CCK8 reagents. **(B)** EdU incorporation assay **(C)** AnnexinV PI/FITC staining in normoxic and hypoxic MKN45 and SGC7901 cells treated with either control or shGAPLINC showing reduced proliferation, reduced DNA replication and increased apoptosis following GAPLINC depletion. The effect was more obvious under hypoxia (^*^P < 0.05, ^**^P < 0.05).

The results above prompted us to examine the effect of the HIF-1α-GAPLINC axis on tumor growth. GAPLINC was knocked down in MKN45 and SGC7901 cells using two shRNAs (Figure 3). We next assessed cell proliferation, migration, invasion, and apoptosis rate. The CCK8 assay was performed out to examine the functional role of GAPLINC in cell proliferation. Cells were transfected with GAPLINC shRNA or empty vector. Under normoxic conditions (Figure 4A), cell proliferation was decreased in the shGAPLINC group compared with that in the shGAPLINC-NC group in the MKN45 and SGC7901 cell lines (*P* < 0.05). This suggested that GAPLINC is a carcinogenic factor involved in regulating GC viability. Under hypoxia, the viability of GC cells was inhibited; however, the shGAPLINC group showed a lower survival rate than the shGAPLINC-NC group (*P* < 0.01), suggesting that GAPLINC plays an important role in sustaining cell survival under hypoxia. EdU incorporation assay results supported this phenomenon (Figure 4B). The proportion of positive cells (green) among total cells (blue) represented the mitotic cell population. Positive cell numbers were lower in shGAPLINC groups than shGAPLINC-NC groups (*P* < 0.05).

GAPLINC played a vital role in inhibiting cell apoptosis according to the cell apoptosis assay (Figure 4C). The shGAPLINC groups showed higher apoptosis rates than the shGAPLINC-NC groups under normoxia or hypoxia. Under hypoxic conditions, GAPLINC knockdown resulted in more cell death in MKN45 cells (*P* < 0.01).

**Figure 5:**
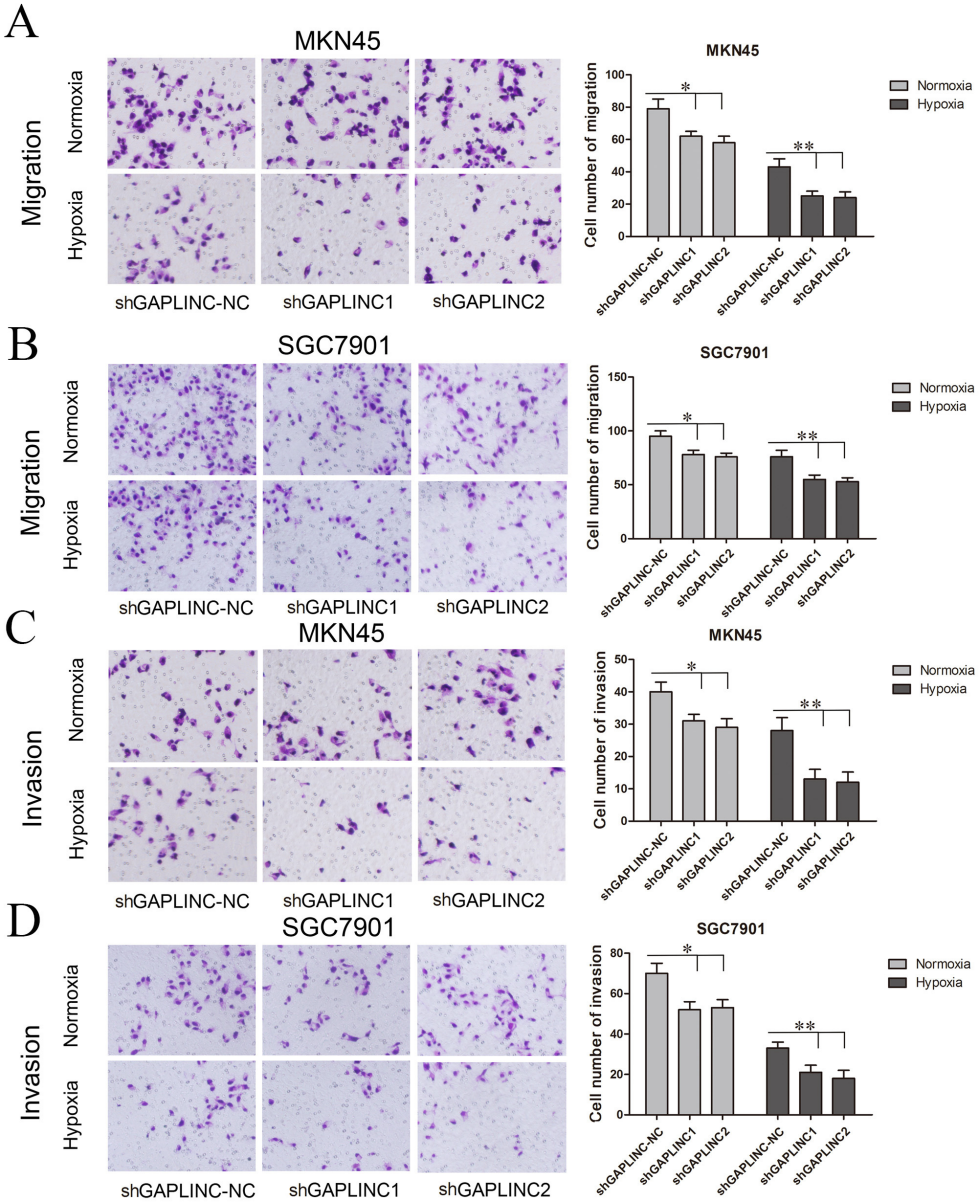
**GAPLINC promoted tumor cell migration and invasion abilities of MKN45 and SGC7901 cells especially under hypoxic conditions**. Values represent the mean ± SD. **(A,B)** Cell migration abilities under nomoxia and hypoxia. **(C,D)** Cell invasion abilities under nomoxia and hypoxia (^*^P < 0.05, ^**^P < 0.05).

The cell migration and invasion assays showed similar results (Figures 5A–D and Figure S1). GAPLINC knockdown attenuated cell mobility in 12 and 48 h after seeding. According to the results, cell migration and invasion abilities relied more on GAPLINC under hypoxia than normoxia. These results clearly revealed that GAPLINC could sustain cell survival especially under poor aerobic conditions.

### HIF-1α transcriptionally activated GAPLINC

**Figure 6:**
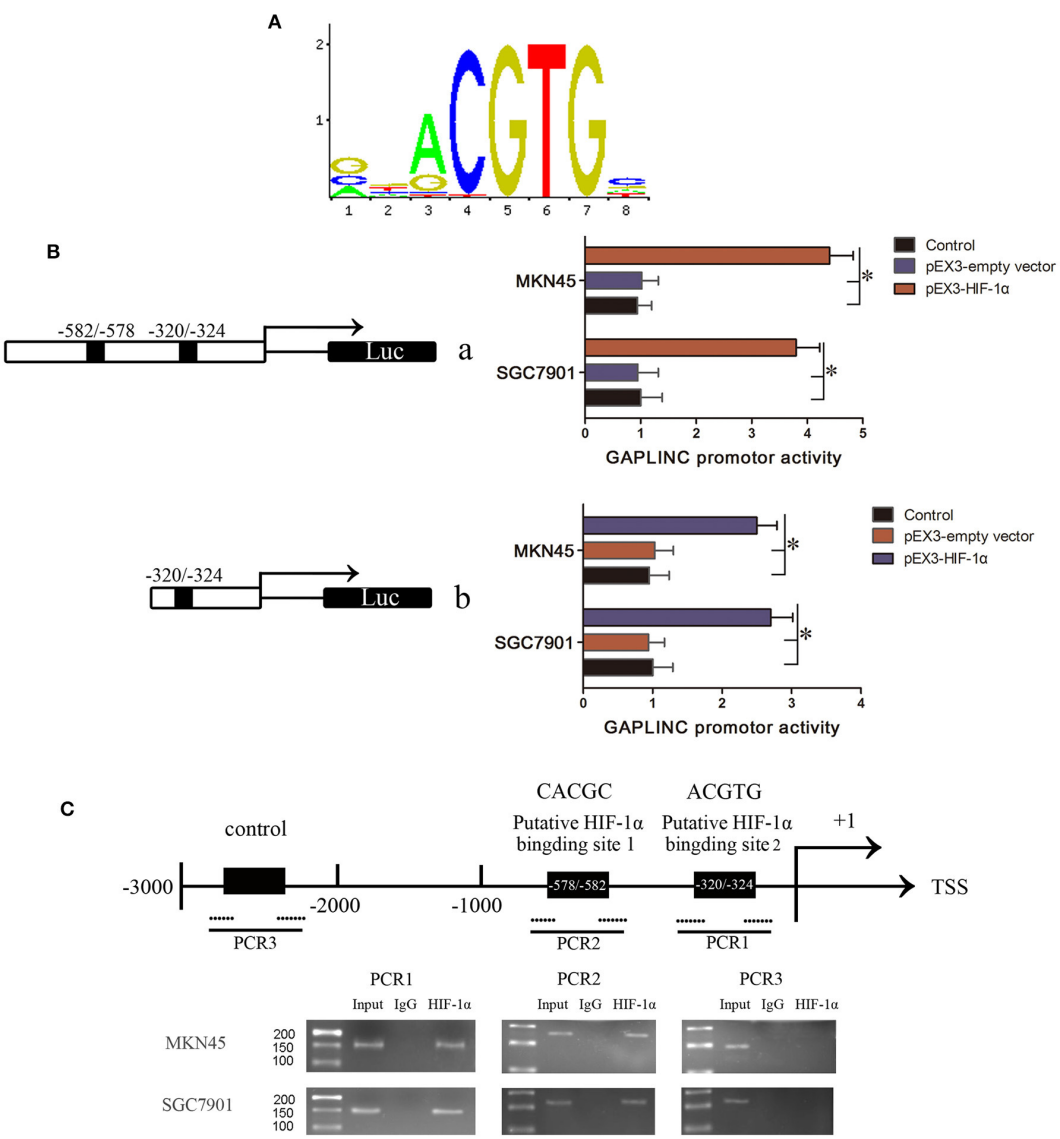
**HIF-1α bound to the HRE of GAPLINC and triggered transcriptional activation. (A)** The binding motif of HIF-1α transcription pathway. **(B)** Effect of HIF-1α on the promoter activities of GAPLINC in MKN45 and SGC7901 cells. Schematic depiction of the different reporter constructs (a and b) used and the luciferase activity. Plasmid activity after normalization with the co-transfected reference vector pRL-TK is shown in the X-axis. Background luminescence from cells transfected with empty pGL3-basic vector was substracted from the sample readings. **(C)** The ChIP assay indicated that HIF-1α binds to the GAPLINC promoter in MKN45 and SGC7901 cells. Transcription start site (TSS) and putative HIF-1α binding sites are indicated. Immunoprecipitated DNA was amplified by PCR. Normal rabbit IgG was used as a negative control. Visualized bands were considered as positive results and indicated endogenous binding of HIF-1α to DNA fragments (^*^P < 0.05).

We speculated that there may be a direct interaction between GAPLINC and HIF-1α, the most important factor induced by hypoxia. HIF-1α binds the “ACGTG” motif according to JASPAR prediction (Figure 6A) and the “CACGC” sequence (Ren et al., 2014), which is characteristic of hypoxic response elements. Screening of the promoter region of GAPLINC identified two potential binding regions for HIF-1α (−320/−324 and −578/−582). To further investigate the mechanism underlying the HIF-1α mediated regulation of GAPLINC expression, a luciferase assay was performed (Figure 6B). The position of the transcription start site (TSS) was predicted by DBTSS. Putative and wild-type HIF-1α binding sites were constructed. Cotransfection with pEX3-HIF-1α resulted in the activation of the GAPLINC promoter containing two HREs by 4.3- and 3.7-fold in MKN45 and SGC7901 cells, respectively. For the GAPLINC promoter containing one HRE, the activation was 2.5- and 2.8-fold in MKN45 and SGC7901 cells, respectively.

To confirm that HIF-1α binds to the HREs of the GAPLINC promoter, a ChIP assay was performed (Figure 6C). Pull down with an anti-HIF-1α antibody identified the PCR fragments of two HREs in the GAPLINC promoter. No PCR fragment was detected in samples pulled down by control IgG antibody. No interaction with the negative control region was observed. Taken together, the results of luciferase and ChIP assays demonstrated that HIF-1α could bind to the GAPLINC promoter and upregulate the promoter activities in human MKN45 and SGC7901 cells.

### GAPLINC affected apoptotic and motility related proteins and was induced by hypoxia in xenograft tumors

**Figure 7:**
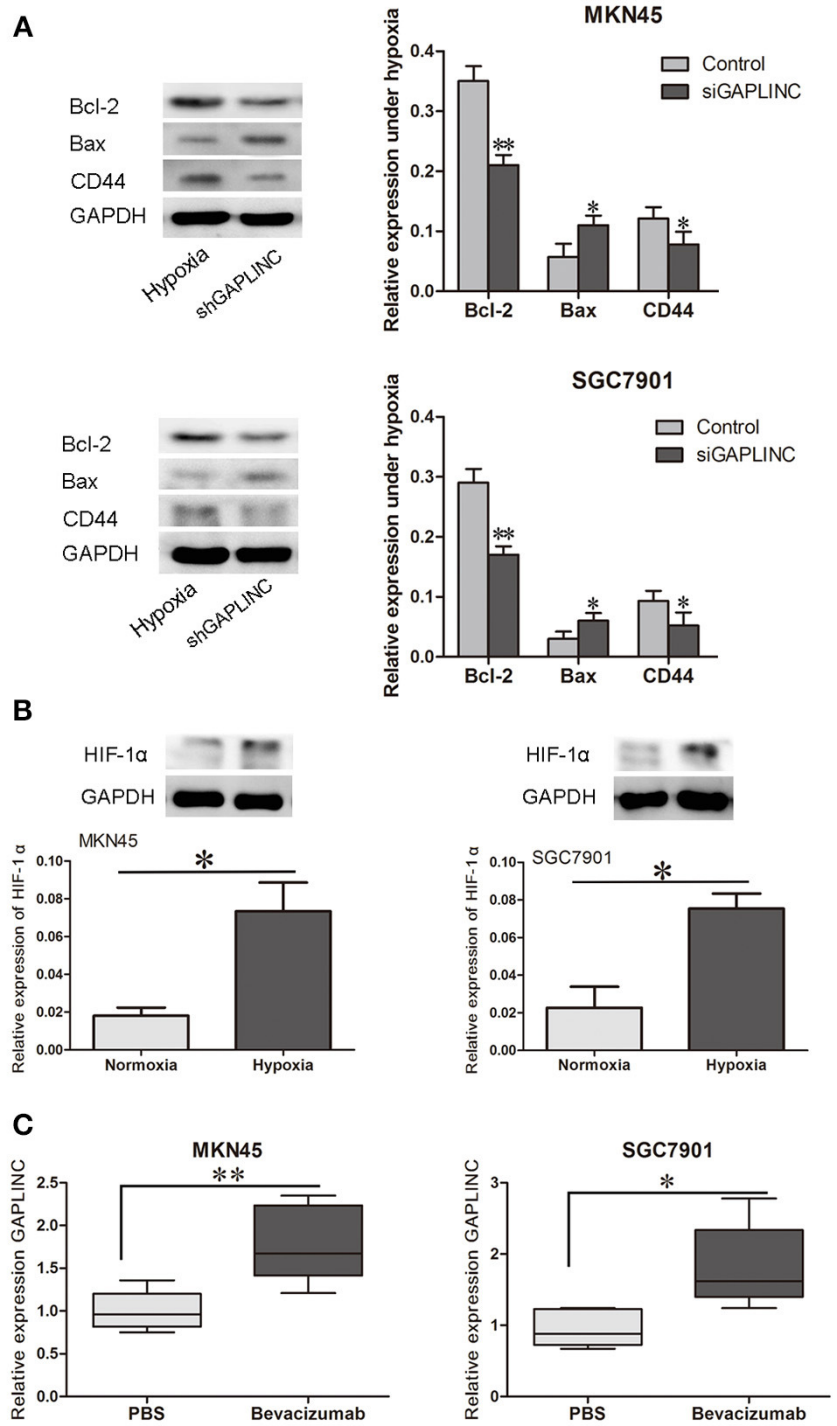
**GAPLINC regulated tumor-related proteins under hypoxia and their levels were increased in solid tumors under simulated hypoxic conditions. (A)** GAPLINC ablation upregulated Bcl-2, CD44, and TGFBR2 and downregulated Bax in MKN45 and SGC7901 cells under hypoxia (^*^P < 0.05 vs. the respective control group). **(B)** Nude mice were treated with the anti-angiogenesis agent bevacizumab or vehicle only. HIF-1α was induced in tumor xenografts treated with bevacizumab. **(C)** GAPLINC expression was upregulated in the bevacizumab group compared with the PBS groups (^*^P < 0.05, ^**^P < 0.05).

Despite reports that GAPLINC increases GC invasiveness by rescuing CD44 from miR-211, we next examined functional proteins related to tumor growth and invasiveness. Since GAPLINC can absorb miR-211, which increases Bcl-2, and Bcl-2 abrogation upregulates the apoptotic protein Bax, Bcl-2, and Bax expressions were detected. As shown in Figure 7A, GAPLINC ablation upregulated Bcl-2, increased the CD44 content, and downregulated Bax in MKN45 and SGC7901 cells under hypoxia (*P* < 0.05). This could partly account for the phenomenon that GAPLINC rescues cell viability and invasiveness under poor oxygen conditions.

To further confirm the hypoxic induction of GAPLINC, an *in vivo* xenograft model was used. Nude mice were raised under pathogen free condition, and then MKN45 and SGC7901 cells were inoculated at a density of 10^6^ subcutaneously. Cells then grew as xenografts. Mice were treated with either bevacizumab to increase tumor hypoxia or PBS as vehicle control (Choudhry et al., 2015). As shown in Figure 7, bevacizumab administration upregulated GAPLINC RNA levels in xenografts derived from both MKN45 and SGC7901 cells. As a positive control, HIF-1α levels were measured in xenograft tumors from both cell lines. HIF-1α protein levels were increased after bevacizumab treatment along with GAPLINC. Taken together, these results indicated that GAPLINC was induced by hypoxia both *in vivo* and *in vitro*.

## Discussion

A considerable amount of evidence supports that aberrant expression of lncRNAs is not only involved in the regulation of the eukaryotic genome, but also plays a role in tumor malignancy (Prensner and Chinnaiyan, 2011; Ling et al., 2015; Zhao et al., 2015). The genes with causal roles in GC are usually copy number variations or oncogenic transcription factors (Deng et al., 2012). As a result, tumors are more invasive and uncontrolled. The lncRNA GAPLINC functions as an endogenous molecular sponge by binding to miR-211 with the putative miRNA response element to form a RNA-induced silencing complex (RISC). As a result, CD44 expression is upregulated because of the reduction of free miR-211-3p, which targets CD44 for degradation (Hu et al., 2014). Our research supports a previous report showing that GAPLINC is highly expressed in GC and involved in the pathogenesis of the disease. In the present study, we showed that GAPLINC increased cell viability and decreased cell apoptosis, suggesting that GAPLINC levels are critical for GC cell growth.

GAPLINC promoted cell viability and inhibited cell death under normoxia. Under conditions of hypoxia, GAPLINC strongly promoted cell survival. miR-211, which functions downstream of GAPLINC, downregulates the anti-apoptotic factor Bcl-2 (De Luca et al., 2015). In the present study, knockdown of GAPLINC downregulated Bcl-2 and upregulated Bax. The Bcl-2/Bax ratio, which is described as the “molecular switch” of apoptosis regulation, is the hub of cell death (Chao and Korsmeyer, 1998). GAPLINC silencing decreased theBcl-2/Bax ratio, leading to increased cell death.

Because previous work in normoxia showed that gastric cell migration and invasion abilities are enhanced by GAPLINC, we confirmed that GAPLINC is essential for cell motility under poor oxygen conditions. To exclude the effect of cell number decrease due to apoptosis, we analyzed cell migration and invasion abilities 12 h after seeding (Figure S1). The trend was similar with Figure 5. The attenuation of cell mobility could be partly attributed to the fact that increased GAPLINC can capture miR-211 to rescue CD44 indirectly. CD44 positivity is associated with higher cell mobility, invasiveness, and shorter tumor-free survival, and it promotes the maintenance of stemness in GC cells (Cho et al., 2015). A CD44-SLC1A2 fusion transcript resulting from a paracentric chromosomal inversion establishes a pro-oncogenic metabolic milieu and produces a subtype of chemo-resistant GC cells (Tao et al., 2011).

Generally, solid tumors are characterized by hypoxia in the tumor center, which provides a poor cellular microenvironment with low pH and inadequate supply of nutrients (Pouysségur et al., 2006). Hypoxia in most solid tumors results in an aggressive and invasive phenotype (Ji, 2014) and stem cell-like characteristics (Kim et al., 2009; Zhang et al., 2012). This is partly responsible for resistance to chemotherapy and radiotherapy (Semenza, 2004). Increasing evidence indicates that the expression of certain lncRNAs changes during hypoxia (Yang et al., 2013; Gómez-Maldonado et al., 2015). One outstanding example is lncRNAEFNA3, which is induced by hypoxia and promotes GC cell metastasis and dissemination.

Since the hypoxic condition in GC is general, we explored the regulation and function of GAPLINC under hypoxia. Evidence suggests that some lncRNAs are involved in the HIF transcriptional regulation pathway. NEAT1 is transcriptionally activated by HIF-2 to form nuclear speckles (Choudhry et al., 2015). In the present study, we showed that GAPLINC is upregulated in response to low oxygen, and the modulation of GAPLINC expression was confirmed *in vivo* in the nude mice xenograft models. The anti-angiogenesis drug bevacizumab is used to imitate poor oxygenation conditions, and changes in HIF-1α protein levels indicate hypoxia simulation. We performed qRT-PCR and showed that GAPLINC was upregulated in xenografts of both cell lines. This suggested that GAPLINCis regulated by HIFs. Compared with its expression under normoxia, GAPLINC was downregulated by HIF-1α or HIF-2α ablation under conditions of hypoxia, and HIF-1α knockdown had a greater effect than HIF-2α knockdown. A ChIP assay demonstrated direct binding of HIF-1α to the HREs in MKN45 and SGC7901 cells. Furthermore, a luciferase assay provided supports for the transcriptional regulation. These data indicate that GAPLINC is induced under hypoxic conditions as a target of HIF-1α. In a previous study, HIF-1α was shown to induce GC glycolysis and cell proliferation (Liu et al., 2016); the present study provides additional information about the GC micro environment.

Knockdown of GAPLINC had a greater effect on cell survival and dissemination under hypoxia than under nomoxia. This could be attributed to the fact that GAPLINC is produced in response to stress signals, and knockdown of GAPLINC under hypoxia will lead to a more significant decrease of GAPLINC.

Tumor angiogenesis is essential for the growth of tumors by increasing the delivery of nutrients and oxygen. Therefore, anti-angiogenesis therapy has become a clinical strategy in line with traditional therapies (Ma and Adjei, 2009; Burger et al., 2011). Chemotherapy combined with anti-angiogenesis drugs such as ramucirumab (Javle et al., 2014; Mackey et al., 2015) and apatinib (Li et al., 2013) has shown efficacy in the treatment of advanced metastatic GC. However, hypoxia tolerant cells result in poor treatment effects and prognosis (Bottsford-Miller et al., 2012; Jain, 2014). Therapy targeting HIF and its key downstream signaling pathway may partly reverse this outcome. The present study identified a novel target of the HIF pathway in gastric adenocarcinoma. Our *in vitro* and *in vivo* data demonstrate that GAPLINC contributes to the aggressive phenotype of tumors showing tolerance to hypoxia. These results suggest potential novel therapies, such as gene therapy targeting HIF-1α and GAPLINC. Although, additional studies are needed for the potential clinical application of these strategies, this pathway should be investigated further to provide new options for the treatment of GC.

## Author contributions

LL did cell culture, transfection, and western assay, he wrote most of the manuscript. XHZ did nude mice xenografts and revised the manuscript. RB did rest of the experiments. KY did the statistical calculation. ZT proposed the hypothesis and revised the manuscript and HWZ provided funding support.

## Funding

This work is supported by grants from the Natural Science Foundation of China (nos. 81472806).

### Conflict of interest statement

The authors declare that the research was conducted in the absence of any commercial or financial relationships that could be construed as a potential conflict of interest. The reviewer TL and handling Editor declared their shared affiliation, and the handling Editor states that the process nevertheless met the standards of a fair and objective review.

## Correction note

A correction has been made to this article. Details can be found at: 10.3389/fphys.2026.1785431.
