# Correction: Hypoxia promotes gastric cancer malignancy partly through the HIF-1α dependent transcriptional activation of the long non-coding RNA GAPLINC

**DOI:** 10.3389/fphys.2026.1785431

**Published:** 2026-01-30

**Authors:** Lei Liu, Xihe Zhao, Huawei Zou, Rubing Bai, Keyu Yang, Zhong Tian

**Affiliations:** 1 General Surgery Department, Shengjing Hospital, China Medical University, Shenyang, China; 2 Oncology Department, Shengjing Hospital, China Medical University, Shenyang, China; 3 General Surgery Department, The Forth Hospital, China Medical University, Shenyang, China

**Keywords:** hypoxia, HIF-1α, lncRNA, GAPLINC, gastric cancer

In the published article, there was an error in Figure 5C *normoxia shGAPLINC-NC group* as published. The figure was misused with Figure 5A hypoxia shGAPLINC-NC group. The corrected Figure 5 appears below.

**FIGURE 5:**
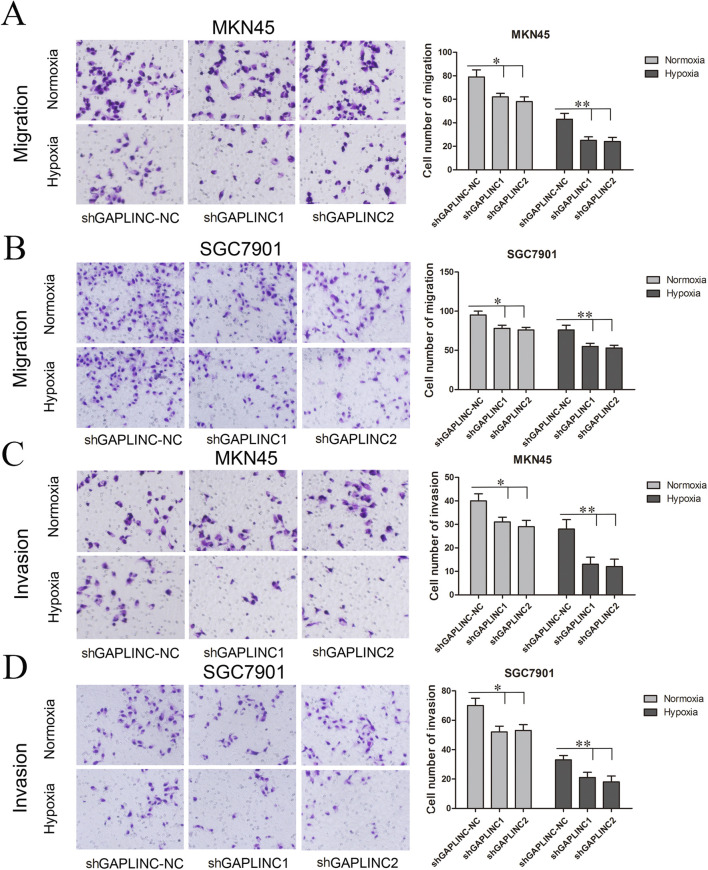
GAPLINC promoted tumor cell migration and invasion abilities of MKN45 and SGC7901 cells especially under hypoxic conditions. Values represent the mean ± SD. **(A,B)** Cell migration abilities under nomoxia and hypoxia. **(C,D)** Cell invasion abilities under nomoxia and hypoxia (*P < 0.05, **P < 0.05).

The original article has been updated.

