# Supplementary figures and images for: Hypoxia Promotes Gastric Cancer Malignancy Partly through the HIF-1α Dependent Transcriptional Activation of the Long Non-coding RNA GAPLINC
Source: Front Physiol. 2016 Sep 27;7:420. doi: 10.3389/fphys.2016.00420 (PMC5037220; doi:10.3389/fphys.2016.00420)

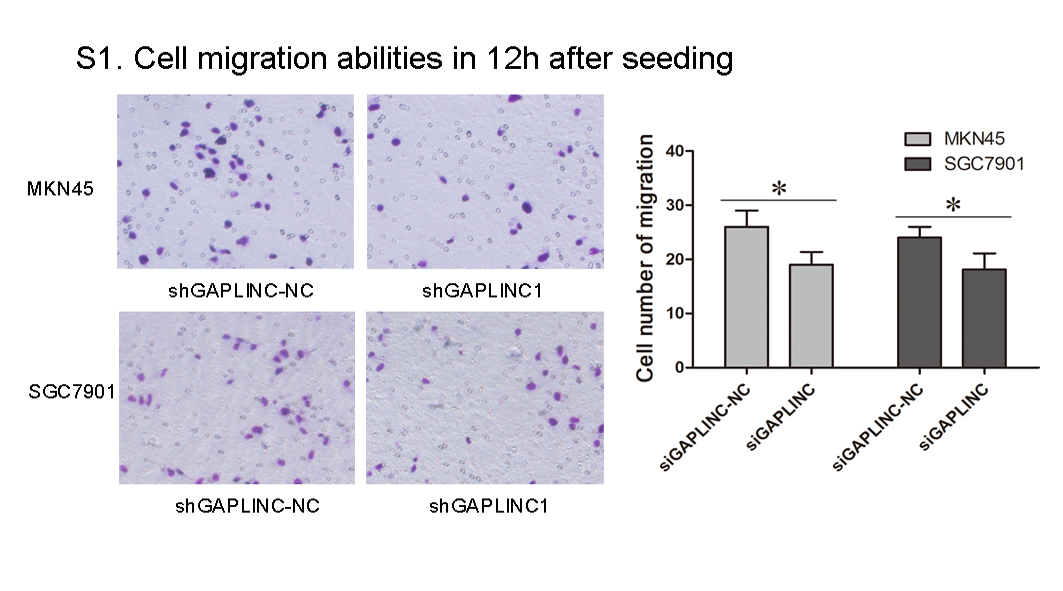

Supplement: Supplementary file 1 [file Image1.TIF]
